# Evaluation of the Vibe Actigraph in Patients With Chronic Obstructive Pulmonary Disease: A Pilot Study

**DOI:** 10.1109/JTEHM.2020.3018399

**Published:** 2020-08-20

**Authors:** Nafeez Syed, Jeremy D. Road, Christopher J. Ryerson, Jordan A. Guenette

**Affiliations:** 1 Centre for Heart Lung InnovationProvidence Health Care Research Institute, The University of British Columbia, St. Paul’s Hospital 8166 VancouverBCV6Z 1Y6Canada; 2 Department of Physical TherapyThe University of British Columbia 8166 VancouverBCV6T 1Z4Canada; 3 Department of PhysiotherapyManipal College of Health ProfessionalsManipal Academy of Higher Education 76793 Manipal576 104India; 4 Division of Respiratory MedicineDepartment of MedicineThe University of British Columbia 8166 VancouverBCV6T 1Z4Canada

**Keywords:** Actigraph, COPD, polysomnography, sensitivity, specificity, validation

## Abstract

Study objective: To validate the Vibe actigraph in assessing sleep-wake patterns compared to polysomnography (PSG) in patients with COPD. Methods: Nine stable COPD patients wore actigraphs while undergoing PSG. The correlation between total sleep time (TST), total sleep period (TSP), sleep onset latency (SOL), wake after sleep onset (WASO), and sleep efficiency was determined for corresponding measurements from the actigraph and PSG. Sensitivity, specificity, and positive and negative predictive values were calculated for the actigraph, considering PSG the gold standard. Levels of agreement between the variables of PSG and the actigraph were estimated using Bland-Altman plots. Results: A strong and statistically significant correlation was noted between PSG and the actigraph in detecting movement during sleep [mean activity score (counts)], TST and TSP (all r_s_ = 0.83; p = 0.005). The median agreement of sleep and wake counts between PSG and the actigraph was 73% and the Cohen’s Kappa value was 0.66. The medians of sensitivity and specificity of the actigraph for detecting sleep versus PSG were 84 and 66%, respectively. The median positive and negative predictive values of the actigraph were 74 and 72%, respectively. Conclusions: This study demonstrated that, under controlled laboratory conditions, the Vibe actigraph in its default settings is a promising tool for the detection of sleep-wake parameters in a small number of ambulatory patients with COPD. Clinical Impact: The actigraph used in this pilot study suggests that these devices could provide clinically relevant information in COPD to better understand the relationship between sleep and health in this population.

## I. Introduction

With over 250 million cases globally, chronic obstructive pulmonary disease (COPD) is projected to be the third leading cause of death in the world by 2030 [1]. Disturbed sleep is a common feature of COPD, being 3-times more prevalent than in the general population [2]. Sleep-related complaints are the third most commonly reported complaint in COPD [3], ranking behind only dyspnea and fatigue [4]. Poor sleep quality predicts subjective health-related quality of life in COPD [5], and is also correlated with disease exacerbations and hospitalization [6]. Inpatient polysomnography (PSG) is the gold standard for sleep assessment and sleep laboratory measurements [7]; however, PSG is intrusive, expensive, and logistically challenging. Given these barriers, PSG is recommended in COPD only if there is suspicion of sleep apnea, complications due to hypoxemia that are unexplained by awake arterial oxygen levels, and the presence of pulmonary hypertension that is out of proportion to the degree of pulmonary function impairment [8].

Actigraphs provide an estimate of time asleep based on the marked decrease in peripheral limb movements that occur during sleep. The correlation coefficients of actigraphs versus PSG for sleep percentage and sleep latency have been reported to be between 0.82-0.89 and 0.92-0.98, respectively, in a variety of populations (e.g. [9], [10]). Although actigraphs have become an important tool for the direct assessment of COPD patients [11], the ability of these devices to measure sleep quality in this population is unclear. Despite an increased effort to use actigraphs to diagnose sleep disturbance in patients with conditions such as COPD, their usage remains controversial because they tend to misclassify quiescent wakefulness as sleep (especially in those with fragmented sleep) [12], and the challenges in optimizing the duration of consecutive immobile and active minutes for sleep onset and end, respectively. Only two studies used an actigraph to either characterize sleep abnormalities in COPD [11], or to customize the scoring settings [13]. They reported that not only is sleep markedly impaired in stable COPD compared to healthy adults, and that the severity of impairment is related to the severity of dyspnea [11], but also the default actigraphy settings may not be optimal for people with COPD [13]. The authors of the latter study compared the sleep characteristics to the gold standard PSG but they did not conduct an epoch-by-epoch direct comparison between methods. The absence of validation studies for most commercially available actigraphs remains one of the most important limitations of this method of assessing sleep [14]. The Vibe actigraph (Agartee Technologies Inc., Vancouver, Canada) is a multi-modal sensor that examines sleep quality, quantity, and activity among other variables such as physical activity, oxygen saturation, air quality, and both maintenance and rescue inhaler usage. In contrast to other actigraphs, the Vibe has been specifically designed to monitor individuals with chronic respiratory conditions, particularly those with COPD. The purpose of this study was to validate the Vibe actigraph in its default settings and assess epoch-by-epoch sleep-wake patterns compared to PSG, alongside estimating its sensitivity, specificity, and predictive ability in COPD patients.

## II. Materials and Methods

### A. Participants

Nine participants with stable COPD were consecutively recruited for this cross-sectional pilot study. The study received institutional ethics approval and informed consent was obtained from all participants. Inclusion criteria were: a diagnosis of COPD grade II to IV according to GOLD criteria [15], positive smoking history, and COPD stability as assessed by their physician. Participants who had a history of exacerbation ≤4 weeks prior to the study, current treatment with oral steroids, hypnotic-sedative medication, or nocturnal oxygen therapy, those involved in night shift-work, and those with any serious disability apart from COPD were excluded. Participants kept a regular sleep–wake schedule the week prior to the study (≥6.5 h/night) and were asked to refrain from caffeine and alcohol 24 h before the study and throughout the experimental day, but were allowed to take their medications as prescribed by their treating physician. Spirometry was assessed during their visit to the Respiratory Outpatient Clinics as per American Thoracic Society guidelines [16].

### B. Polysomnography (PSG)

PSG was performed in a dedicated inpatient sleep laboratory using conventional instrumentation and analysis according to the recommendations on syndrome definition and measurement techniques published by the American Academy of Sleep Medicine [17]. Participants arrived at the sleep laboratory between 19:30–20:00 hours and were in bed by 22:30 hours. Participants were instructed to follow their regular pre-sleep activities and fall asleep as per their usual sleeping habits. Data were interpreted using 30-sec scoring epochs and the sleep staging and arousals were scored as per standard recommendations [17].

### C. Vibe Actigraph

The Vibe actigraph device (Agartee Technology Inc., Vancouver, Canada) was worn on each wrist throughout the night of the PSG in order to monitor sleep-wake events. The motion sensors of the actigraph captured accelerations of the wrists, measured as activity counts using a sensitivity threshold of zero. As these activity counts are collected based on high-resolution acceleration signals, they are considered a good and reliable measure to estimate movement [18]. The proprietary algorithms were initially developed from the work of Sadeh *et al.* [19] and de Souza *et al.* [20] and subsequently modified using tree based machine learning and based on pilot testing in individuals with COPD. A summary report containing parameters of sleep-wake periods, their timing, duration, and other characteristic details derived using the device’s default settings were then compared to those of PSG.

### D. Data Collection

The actigraph on the non-dominant wrist was used for all analyses whereas the actigraph on the dominant wrist was used as a backup in case of technical difficulties. Harmonization of PSG and the actigraph was achieved by synchronizing the time stamps of the actigraph to that of the computer clock used for PSG data collection at lights out. Participants were allowed to determine lights out based on their personal preference. An experienced sleep technician, blinded to the actigraph data, monitored and scored the PSG sleep data. The investigator analyzing and summarizing the actigraph data was blinded to the PSG data. The actigraphs and PSG electrodes were disconnected when participants spontaneously woke up the following morning (06:00- 07:30 hours). The following sleep parameters were compared between the actigraph and PSG: sleep onset time (lights off), sleep offset time (lights on), total sleep time (TST) scored during the total recording time, duration of time spent in sleep from sleep onset to the last epoch of sleep (i.e., total sleep period, TSP), sleep onset latency (SOL), wake-after-sleep-onset (WASO), number of awakenings (NOA), and sleep efficiency.

### E. Questionnaires

The severity of dyspnea was quantified using the modified Medical Research Council (mMRC) dyspnea scale [21] and the impact of COPD symptoms on health status was estimated using the COPD Assessment Test (CAT) [22]. Sleep quality and other sleep-related characteristics over the preceding one-month were collected using the Pittsburgh Sleep Quality Index (PSQI) questionnaire. The Epworth Sleepiness Scale (ESS) was used to evaluate daytime somnolence [23].

### F. Statistical Analysis

All analyses were conducted using Python (Python Software Foundation Inc., USA) and SPSS 20.0 (SPSS Inc, Chicago, IL). For all analyses, sleep data from both PSG and the actigraph were reduced to binary form (0 = any stage of sleep, 1 = wakefulness). Spearman’s correlation coefficients were calculated to evaluate the relationship between actigraph measures and PSG. Additionally, we tested sleep-wake events during individual epochs of the PSG and the actigraph to derive estimates of agreement on true sleep (TS), false sleep (FS), true awake (TA), and false awake (FA) [24]. We also calculated Cohen’s kappa based on epoch-by-epoch comparisons to evaluate the agreement with PSG beyond what could be due to chance. A repeated measures ANOVA with sleep condition and scoring method (PSG and Vibe) factors were performed on wake status. Mauchly’s test indicated that the assumption of sphericity was met, therefore degrees of freedom were corrected using Greenhouse-Geisser estimates of sphericity ($\varepsilon =1.0$). Sensitivity, for the purposes of this study, was defined as the ability of the actigraph’s algorithm to detect sleep, whereas specificity referred to its ability to detect wakefulness. Epoch-by-epoch concordance was then calculated to estimate the agreement, along with sensitivity and specificity, respective measures reflecting the ability of how accurately the actigraph is in detecting sleep and wake events compared to PSG. We also calculated the positive, and negative predictive values within each individual participant to estimate the probability of match between the actigraph and PSG for TS, FS, TA, and FA events [24]. Data are reported as median (IQR) unless otherwise specified. The level of significance was set at p < 0.05.

## III. Results

A total of nine individuals with COPD were included (Table 1). Four participants had moderate (Grade II) and the remaining had severe (Grade III) COPD, with all participants reporting regular use of inhaled medications. Subjective sleep quality assessment showed that all participants were poor sleepers (median Global PSQI score 7 (3) [range: 6 to 11]). The median ESS score was 6 (6) (range: 1 to 12), with five participants reporting getting enough sleep and two reporting excessive day-time somnolence.

**TABLE 1:** Demographic Characteristics of the Study Participants (n = 9)

Parameter	Median (IQR)
Sex (Male/Female)	5/4
Age (years)	72 (16)
BMI (kg/m^2^)	30 (9)
FVC (%predicted)	70 (19.5)
FEV_1_(%predicted)	48 (11)
FEV_1_/FVC (%)	48 (16)
FEF_25-75_ (%predicted)	18 (9)
PEF (%predicted)	47 (19)
mMRC (0–4)	2 (2)
BDI focal score	6 (4)
Magnitude of task	2 (1)
Magnitude of effort	2 (1.5)
Functional impairment at home	3 (1)
Functional impairment at work	0 (2)
CAT	21 (11)
ESS	6 (6)
PSQI Global score	7 (3)
AHI	7 (17)
COPD grade (GOLD II/III/IV)	4/5/0

BMI, body mass index; FVC, forced vital capacity; FEV_1_, forced expiratory volume in 1 second; FEF_25-75_, forced expiratory flows between 25 and 75% of forced vital capacity; PEF, peak expiratory flow; mMRC, modified medical research council scale; BDI, baseline dyspnea index; CAT, COPD Assessment Test; ESS, Epworth Sleepiness Scale; PSQI, Pittsburgh Sleep Quality Index; AHI, apnea hypopnea index; GOLD, Global Initiative for Chronic Obstructive Lung Disease.

Sleep-wake variables detected using PSG and the actigraph are summarized in Table 2 along with the correlation coefficients and 95% CI. There was a strong and statistically significant correlation noted between PSG and the actigraph in sleep counts (r_s_ = 0.83, p = 0.005), TST (r_s_ = 0.83, p = 0.005), and TSP (r_s_ = 0.83, p = 0.005). Moderate non-significant correlations were found in awake counts (r_s_ = 0.55), sleep efficiency (r_s_ = 0.55), SOL (r_s_ = 0.50) and number of awakenings (r_s_ = 0.50).

**TABLE 2:** Correlation Between the Sleep and Wake Characteristics of PSG and Actigraph

Variable		Median (IQR)	r	p
Wake Counts	PSG	372 (259)	0.55	0.12
	ACTI	297 (189)		
Sleep Counts	PSG	480 (379)	0.83	$0.005\ast $
	ACTI	586 (222)		
TST (min)	PSG	240 (190)	0.83	$0.005\ast $
	ACTI	293 (111)		
TSP (min)	PSG	443 (165)	0.83	$0.005\ast $
	ACTI	445 (105)		
Sleep efficiency (%)	PSG	56 (34)	0.55	0.12
	ACTI	71 (20)		
WASO (min)	PSG	137 (73)	0.20	0.61
	ACTI	143 (111)		
SOL (min)	PSG	42 (36)	0.50	0.90
	ACTI	4 (4)		
NOA	PSG	24 (11)	0.50	0.17
	ACTI	19 (9.5)		

ACTI, actigraph; PSG, polysomnography; TST, total sleep time; TSP, total sleep period; WASO, wake after sleep onset; SOL, sleep onset latency; NOA, number of awakenings; $\ast \text{p} < 0.05$

**FIGURE 1.**
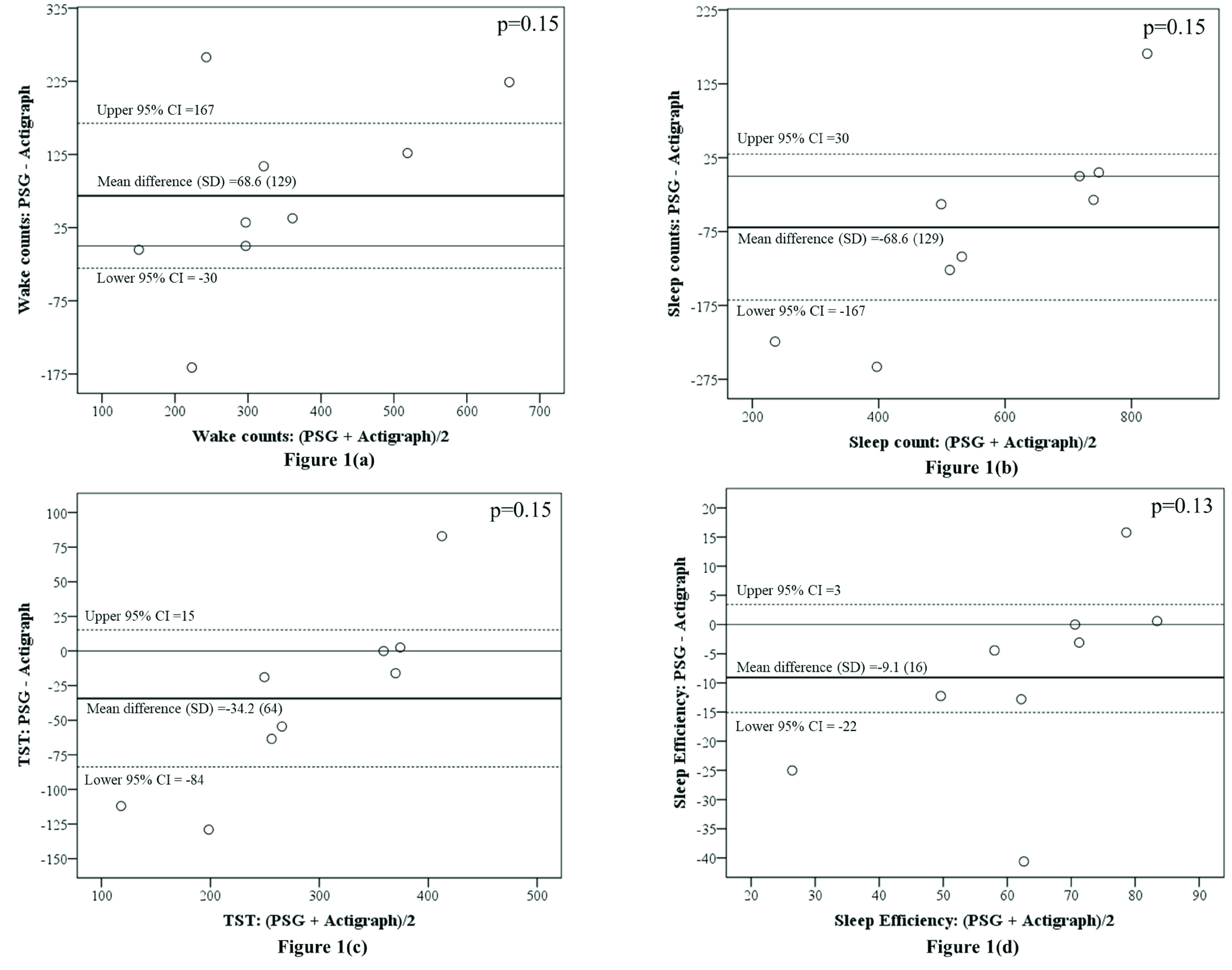
(a) to (d). Bland-Altman plots for comparability between wake counts, sleep counts, total sleep time (TST), and sleep efficiency estimated using PSG and actigraph. $^\ast \text{p} < 0.05$.

Bland-Altman plots were used to assess the comparability and to define the intervals of agreement between the actigraph and PSG (**Fig. 1a-d**). Results of these plots revealed no significant difference between PSG and actigraphy in wake counts, sleep counts, TST and sleep efficiency, and the estimates of the comparability are displayed in Table 3. However, visual inspection of the Bland-Altman plots shows that PSG estimated wake more than that of the actigraph (**Fig. 1a**) and the agreement between PSG and the actigraph decreased as the average wake counts increased. PSG estimated sleep counts less than the actigraph (**Fig. 1b**), but the agreement between PSG and the actigraph appeared to increase as the average sleep counts increased. The actigraph estimated TST (**Fig. 1c**) and sleep efficiency (**Fig. 1d**) greater than PSG, and the agreement between PSG and the actigraph appeared to also increase as the average TST and sleep efficiency increased.

**TABLE 3:** Estimates of Comparability Between PSG and Actigraph

Parameter	Mean difference	SD	95% CI	p
Wake counts	68.6	129	−30 to 167	0.15
Sleep counts	−68.6	129	−167 to 30	0.15
TST	−34.2	64	−84 to 15	0.15
Sleep Efficiency	−9.1	16	−22 to 3	0.13

SD, Standard deviation; TST, total sleep time; CI, confidence interval; $\ast \text{p} < 0.05$

Cohen’s Kappa estimates of agreement and the positive, and negative predictive values for TA, FA, TS, and FS measured by PSG and the actigraph are shown in Table 4. The unweighted medians of agreement and Cohen’s Kappa between PSG and the actigraph were 73%, and 0.66 respectively. The medians of sensitivity and specificity of the actigraph to detect sleep in comparison to PSG were 84 and 66%, respectively. The positive and negative predictive values of the actigraph were 74 and 72%, respectively. Analysis of the specificity data showed a significant main effect for device type (p < 0.001) in detecting wake status of the participants. The weighted means from the repeated measures ANOVA are shown in Table 4.

**TABLE 4:** Estimates of Agreement for Select Sleep Parameters Between PSG and Actigraph

Participant	PSG vs. Actigraph counts				Agreement (k)	Sensitivity (%)	Specificity (%)	PPV (%)	NPV (%)
	TA	FA	TS	FS					
1	220	77	641	77	0.69	89	74	89	74
2	532	14	110	238	0.70	89	69	32	97
3	386	69	380	196	0.66	85	66	66	85
4	118	163	561	195	0.30	77	38	74	42
5	105	48	703	43	0.67	94	71	94	69
6	68	46	222	304	0.17	83	18	42	60
7	263	79	401	117	0.66	83	69	77	77
8	192	75	402	184	0.51	84	51	69	72
9	83	223	685	57	0.31	75	59	92	27
Unweighted Median	192	75	402	184	0.66	84	66	74	72
Weighted Mean (±) SD	222 ± 145	93 ± 63	471 ± 193	150 ± 80	0.52 ± 0.2	84 ± 6	58 ± 16	71 ± 20	66 ± 21

TA, True awake; FA, false awake; TS, true sleep; FS, false sleep; PPV, positive predictive value; NPV, negative predictive value; k, Cohen’s Kappa SD, Standard deviation; TST, total sleep time; CI, confidence interval; $\ast \text{p} < 0.05$

## IV. Discussion

This is the first study to compare the accuracy of an actigraph in its default settings to PSG in detecting sleep-wake events using epoch-by-epoch direct comparison in participants with moderate-to-severe COPD. Our findings suggest that the Vibe actigraph is a promising tool for detecting sleep-wake events in this population even in its default settings. In addition to detecting sleep events (e.g., TST, sleep efficiency) with high sensitivity, the Vibe actigraph appears to be more specific in differentiating wake events in participants with COPD compared to actigraphs used in other populations.

The main findings of this pilot study are consistent with previous studies showing strong correlations between actigraphy and PSG for differentiating sleep from wake, albeit in other clinical conditions [25]. Sensitivity of the Vibe actigraph to identify sleep accurately was found to be 84% versus PSG. This level of sensitivity is lower than that reported previously in other populations (e.g. infants [26], young children [27], and healthy volunteers [20]) where values ranged from 92-97%. The discrepancy may be attributable to differences in populations, duration of data recordings, and the methods used for identifying sleep periods.

Unlike the present study, previous studies reported the sensitivity of data collected from sleep diaries and actigraphs, not from PSG, over multiple days (e.g., 1–23 days) and with variable periods of data recording per night [28]. Moreover, the duration of data recording using an actigraph demonstrated that acceptable data-stability levels for TST and percent sleep efficiency occurred only after 7 nights of recording [29]. Nevertheless, using data collected over only one night, the present actigraph showed a greater correlation with PSG in detecting sleep counts along with TST and TSP, in comparison to other actigraphs used to study a variety of populations [25], [27], [30]. Therefore, the Vibe actigraph could potentially be used as a supplement to PSG, for measuring sleep events in free-living conditions, with minimal impact on one’s sleep pattern. Nevertheless, since a significant correlation with PSG was not noted in all of the sleep-wake outcomes, similar to that reported by Kapella *et al.* [13], future work is needed in a large sample to determine how the Vibe actigraph performs with customized settings and over multiple nights. The mean specificity of the Vibe actigraph was 58% and was significantly different from PSG. The specificity was similar to that reported by Insana *et al*. [26] in healthy infants (59%) but higher than that reported in children (24%) [27], healthy volunteers (35%) [31], and patients with obstructive sleep apnea (48%) [32]. Although the better specificity could potentially address a major limitation posed in using actigraphs as alternatives to PSG by minimizing the risk of estimating quiescent wakefulness as sleep, it is still suboptimal and indicates the need for future research.

The mean agreement of the Vibe actigraph with PSG in this study was found to be 73± 11% in detecting sleep accurately. Although the agreement rates were in line with some studies [33], it is lower than that reported by others (78% to 97%) [34]. The higher rates of agreement reported in the aforementioned studies could be because they studied either children [27], healthy individuals [31], insomniacs [33], or patients with psychiatric disorders [34], rather than those with COPD. Findings of this study are similar to those reported by Mullaney *et al.* [35] who also noted that epoch-by-epoch actigraph-PSG agreement was significantly lower in elderly patients (>50 yrs) and in short sleepers (< 390 minutes), as compared to healthy individuals. It is known that there is a natural tendency for older individuals to be light sleepers and be more restless during sleep [36], which could have led to the lower agreement rates in this study. Given that most of these features are commonly observed in people with COPD, it seems reasonable to speculate that the lower agreement rate can be attributed, at least in part, to the study group investigated. The mean TST of the COPD participants in this study was 272 min and 306 min as estimated by PSG and actigraphy, respectively. Only one participant in the study sample had a PSG reported TST of greater than 390 min (i.e., 454 min).

The lower TST noted could be attributed to the lower sleep quality in COPD, which also decreases the accuracy of the algorithms used in activity monitors [32]. Therefore, in line with the recommendations of Sadeh *et al*. [37] the threshold of the study actigraph was adjusted to optimally balance both sensitivity and specificity and also to have the best agreement with PSG in detecting both sleep and wake patterns. This could have also contributed to a moderate agreement of the actigraph with PSG. Bland-Altman analysis revealed no difference between actigraphy and PSG in wake counts, sleep counts, TST and sleep efficiency, which is in line with the findings of Kapella *et al.* [13] in COPD.

The study actigraph also demonstrated good positive (71%) and negative (66%) predictability of sleep compared to PSG. Although the positive predictive values were considerably lower than those reported by So *et al.* (2005) [38] [96.5 to 98%] in infants, our negative predictive values were higher (17.0 to 43.6%), indicating that the Vibe actigraph may be more specific than other actigraphs in detecting wake. Despite multiple reports indicating the possibility of impaired sleep in COPD [39], [41], Nunes *et al*. [11] were the first to objectively compare the sleep-wake events between ambulatory COPD patients and their healthy counterparts. Although the authors confirmed that sleep is impaired in COPD, they did not report the validity of the actigraph used in their study. Since the validity of actigraphs can be compromised in special populations [37], and were shown to be reliable only in the elderly or those with insomnia [30], [37], our study aimed to concurrently investigate actigraphy versus PSG in individuals with COPD using a device specifically designed for this population.

This study demonstrated that COPD participants with moderate-to-severe disease have disturbed sleep due to respiratory causes, as shown by the median AHI score of 7 on PSG. The relatively small mean difference of 9% in estimating sleep efficiency indicates that the Vibe actigraph may be a useful tool to estimate disturbed sleep in ambulatory patients with COPD and may help practitioners overcome barriers of using actigraphs in clinical practice to assess sleep in this population [11]. However, our findings must be interpreted cautiously given the relatively small sample size of this pilot study. In addition to our small sample size, we must acknowledge several other limitations of this study. For example, we did not screen participants for obstructive sleep apnea. We also did not include a familiarization night for being monitored in a sleep laboratory, which could have influenced our results. Finally, we included older adults with a relatively high BMI. While this is typical of individuals with COPD, more studies are needed to examine the performance of actigraphy in a more heterogeneous group of individuals with COPD including a wider range of ages, BMI, and levels of disease severity.

In conclusion, this pilot study evaluated the validity of an actigraph concurrently with PSG in ambulatory COPD participants under controlled laboratory conditions. Overall, our preliminary results suggest that the actigraph used in this study is a promising tool for detection of sleep-wake events in its default settings, recognizing that additional studies in different and larger populations are warranted to validate and expand upon these findings. The validity of the device coupled with the fact that actigraphs can provide additional information, suggests that these devices could provide clinically relevant information in this population. Indeed, sleep disorders are a highly prevalent comorbidity in individuals with COPD [42]–[44] and a growing body of literature suggests that disturbed sleep is associated with increased acute exacerbations [44], increased absenteeism [45], daytime sleepiness [41], reduced survival [6], and impaired quality of life in COPD [41]. Actigraphy enables the characterization of sleep and circadian patterns and can be used to document treatment responses [46]. These applications may ultimately prove useful for managing individuals with COPD to improve sleep and quality of life, although this requires further investigation.

Future studies are needed to directly evaluate the performance of the Vibe Actigraph compared to other commercially available devices for monitoring sleep in patients with COPD. Additional studies should also determine the value of tracking sleep over longer periods of time and in response to both pharmacological and non-pharmacological interventions in COPD using the Vibe actigraph. Finally, combining longer term monitoring of sleep with other clinically relevant outcome measures (e.g. physical activity, inhaler use, oxygen saturation, etc.) may provide clinically relevant information to help in the management of COPD.

## Disclosure

*Conflict of Interest:* NS and CJR do not have any conflicts of interest to report. JAG and JDR have received research funding from Agartee Technology Inc. In addition, JDR, JAG, and JAG’s spouse serve as consultants to Agartee Technology Inc. and hold shares in the company.
